# A deep learning approach versus expert clinician panel in the classification of posterior circulation infarction

**DOI:** 10.1016/j.nicl.2025.103732

**Published:** 2025-01-12

**Authors:** Leon S. Edwards, Milanka Visser, Cecilia Cappelen-Smith, Dennis Cordato, Andrew Bivard, Leonid Churilov, Christopher Blair, James Thomas, Angela Dos Santos, Longting Lin, Chushuang Chen, Carlos Garcia-Esperon, Kenneth Butcher, Tim Kleinig, Phillip MC Choi, Xin Cheng, Qiang Dong, Richard I. Aviv, Mark W. Parsons, on behalf of the INSPIRE Study Group

**Affiliations:** a Department of Neurology and Neurophysiology, Liverpool Hospital, Sydney, NSW, Australia; b South Western Sydney Clinical School, University of New South Wales, Sydney, NSW, Australia; c Ingham Institute for Applied Medical Research, Sydney, NSW, Australia; d Melbourne Brain Centre at the Royal Melbourne Hospital, University of Melbourne, Parkville, Australia; e Department of Neurology, John Hunter Hospital, Newcastle, NSW, Australia; f Hunter Medical Research Institute and University of Newcastle, Newcastle, NSW, Australia; g Prince of Wales Clinical School, University of New South Wales, Sydney, NSW, Australia; h Department of Neurology, Royal Adelaide Hospital, Adelaide, SA, Australia; i Department of Neurosciences, Box Hill Hospital, Eastern Health Clinical School, Monash University, Melbourne, VIC, Australia; j Department of Neurology, Huashan Hospital, Fudan University, Shanghai, China; k Division of Neuroradiology, Department of Radiology, University of Ottawa and The Ottawa Hospital, ON, Canada

**Keywords:** Ischaemic stroke, CT perfusion, Deep learning, Posterior circulation stroke

## Abstract

**Background:**

Posterior circulation infarction (POCI) is common. Imaging techniques such as non-contrast-CT (NCCT) and diffusion-weighted-magnetic-resonance-imaging commonly fail to detect hyperacute POCI. Studies suggest expert inspection of Computed Tomography Perfusion (CTP) improves diagnosis of POCI. In many settings, there is limited access to specialist expertise. Deep-learning has been successfully applied to automate imaging interpretation. This study aimed to develop and validate a deep-learning approach for the classification of POCI using CTP.

**Methods:**

Data were analysed from 3541-patients from the International-stroke-perfusion-registry (INSPIRE). All patients with baseline multimodal-CT and follow-up imaging performed at 24–48 h were identified. A cohort of 541-patients was constructed on a 1:3 POCI-to −reference-ratio for model analysis. A 3D-Dense-Convolutional-Network (DenseNet) was trained to classify patients into POCI or non-POCI using CTP-deconvolved-maps. Six-stroke-experts also independently classified patients based upon stepwise access to multimodal CT (mCT) data. DenseNet results were compared against expert clinician results. Model and clinician performance was evaluated using area-under-the-receiver-operating-curve, sensitivity, specificity, accuracy and precision. Clinician agreement was measured with the Fleiss-Kappa-statistic.

**Results:**

Best mean clinician diagnostic accuracy, sensitivity and agreement was demonstrated after review of all mCT data (AUC: 0.81, Sensitivity: 0.65, Fleiss-Kappa-statistic: 0.73). There was a spectrum of individual clinician results with an AUC-range of 0.73–0.86. Best DenseNet performance was recorded with an input combination of NCCT and delay-time maps. The DenseNet model was superior to the best mean clinician performance (AUC: 0.87) and was due to enhanced sensitivity (DenseNET: 0.77, Clinician: 0.65). The degree to which the DenseNet model outperformed each clinician ranged and was clinician specific (AUC improvement 0.01–0.14).

**Conclusion:**

Comprehensive review of CTP improves diagnostic performance and agreement amongst clinicians. A DenseNet model was superior to best mean clinician performance. The degree of improvement varied by specific clinician. Development of a clinician-DenseNet approach may improve inter-clinician agreement and diagnostic accuracy. This approach may alleviate limited specialist services in resource constrained settings.

## Highlights

• Review of NCCT, CTP parametric and automated core-penumbra summary maps improves diagnostic performance in acute POCI.
• A DenseNet model was superior to 6 stroke neurologists in classifying POCI. The degree of improvement varied by neurologist.
• Developing a hybrid DenseNet clinician approach to imaging may improve clinician agreement and diagnostic accuracy.

## 1 Introduction

Posterior circulation infarction (POCI) is a common, disabling and treatable condition which accounts for 20 % of all acute ischaemic stroke (Schulz and Fischer, 2017, Bamford et al., 1991). The posterior cerebral circulation has unique anatomy and physiology differentiating it from the anterior circulation (Pagola et al., 2011). Symptoms and signs of POCI can be subtle in comparison to anterior circulation stroke. Patients with POCI frequently present with non-specific symptoms including headache, dizziness and gait disturbance (Arch et al., 2016). Widely used clinical screening tools such as the Face, Arms, Speech and Time (FAST) score poorly identify POCI (Paul et al., 2013). Compounding this challenging clinical diagnosis; current guideline based hyperacute neuroimaging techniques (Powers et al., 2018) including MRI (Oppenheim et al., 2000) and CT (Ostman et al., 2020) commonly fail to detect POCI. Timely access to MRI is constrained in the emergency setting due to limited 24/7 availability and inability to be performed in patients with metal foreign bodies such as pacemakers. POCI is frequently misdiagnosed (Tarnutzer et al., 2017) with patients experiencing treatment delays (Sarraj et al., 2015) and worse long term functional outcomes (Kim et al., 2017).

CT perfusion (CTP) has been pivotal to expanding eligibility for thrombolysis (Ma et al., 2019, Thomalla et al., 2018) and endovascular thrombectomy (EVT) (Nogueira et al., 2018, Albers et al., 2018). The principal use of CTP has been for patient selection for hyperacute treatments via rapid determination of infarct core and penumbra. Studies have shown that expert examination of parametric maps enhances diagnostic accuracy of POCI (Ostman et al., 2020, Sharon et al., 2016). However, timely access to specialised stroke care including, MRI and imaging interpretation, is constrained in many settings (Edwards et al., 2020). Automated CT based detection methods for POCI could alleviate these resource gaps. Deep learning is a subset of the field of artificial intelligence and utilises neural networks for automated data analysis and prediction of specific outcomes. These techniques have been broadly applied to medicine and the neurosciences. Specific applications to stroke include MRI based diagnosis (Lee et al., 2020) and outcome prediction (Heo et al., 2019). Convolutional neural networks (CNN) are the principal machine learning method for visual object classification. Dense Convolutional networks (DenseNet) have distinct advantages over traditional CNN’s including superior parameter efficiency, improved computation, higher performance and accuracy (Huang et al., 2017). The DenseNet 121 model has been shown to be effective for medical image classification (Urinbayev et al., 2020) and superior to other deep learning approaches (Lakshmi and Sargunam, 2024). There are no studies which apply deep learning to CTP for the diagnosis of POCI. This study aimed to develop a novel deep learning approach to the automated classification of POCI using a DenseNet 121 architecture.

## 2 Methods

### 2.1 Patient Population and selection

Data were analysed from consecutive patients diagnosed with acute POCI enrolled in the INternational Stroke Perfusion Imaging REgistry (INSPIRE). Patients were recruited from 9 hospitals across Australia, Canada and China between February 2007 and February 2020. Patients had multimodal CT (non-contrast CT, CT angiography and CT perfusion) at acute presentation with follow up imaging (CT or MRI) performed within 7 days post stroke. Patients were treated with acute reperfusion therapies (including thrombolysis and mechanical thrombectomy) according to local eligibility protocols. Modified Rankin Scale (mRS) was documented at 90 days following stroke by an accredited assessor. Written informed consent was obtained from all participants enrolled in the registry. The INSPIRE study was approved by the local hospital ethics committees in accordance with Australian National Health and Medical Research Council guidelines.

Baseline and follow up imaging were assessed and labelled by the INSPIRE imaging panel with each case being analysed by at least 2 experienced readers. Cases with disagreement were resolved via consensus review with a third reader. Labelled imaging was used at the ground truth for further analysis. No member of the INSPIRE panel was part of the expert stroke clinician panel used for further imaging analysis included in this study.

All patients met the following imaging specific criteria including; a baseline CTP with less than or equal to 5 mm resolution and at least 120 mm coverage in the z-direction direction. Patients were excluded due to the following reasons including; no follow up non-contrast CT or diffusion weighted MRI, missing CTP data, inadequate coverage of the posterior circulation and excessive artefact or movement during CTP.

Patients were divided into a POCI and general cohort. Inclusion in the POCI cohort required radiological evidence of a POCI; defined as patients with a complete occlusion on baseline CTA represented by a mTICI of 0 in a branch of the vertebrobasilar circulation (defined as vertebral, basilar or posterior cerebral arteries) and evidence of POCI (Bonkhoff et al., 2021) on follow up imaging with an appropriate clinical presentation. Patients were included in the general cohort if they did not meet the POCI cohort inclusion criteria with either; evidence of non-POCI territory stroke on follow up DWI or a non-stroke defined as no lesion on both baseline CTP and follow up DWI.

### 2.2 Image acquisition

Baseline CT imaging was performed using 64-, 128-, 256- or 320- detector scanners. Axial z-axis coverage ranged from 120 mm to 160 mm. Scan acquisition sequence was; a non-contrast helical CT (NCCT) from skull base to vertex followed by CTP and then CTA from aortic arch to vertex. Details of each scanner and CTP protocol are provided in Sup. Table S1.

Follow up MRI was performed ideally at 24 to 48 h post stroke irrespective of treatment using 1.5 to 3 T scanners. The MRI protocol included an axial gradient-echo T2*-weighted series, isotropic diffusion-weighted imaging (DWI), MR time of flight angiography and fluid-attenuated inversion recovery imaging. In instances where an MRI was not possible, a NCCT was performed.

### 2.3 CTP post-processing

CTP maps were processed using a commercial imaging package (AutoMiStar, Apollo medical imaging technologies, Melbourne, Australia). This software automatically performs motion correction. It then automatically derives arterial input and venous output functions by selecting an unaffected major artery (commonly the anterior cerebral artery) and venous sinus (commonly the superior sagittal sinus). Selected inputs were confirmed by an expert analyst prior to image processing. Areas of gliosis, chronic infarction and cerebrospinal fluid were automatically masked from perfusion maps using a Hounsfield unit threshold. CTP source imaging was processed using delay and dispersion corrected singular value decomposition deconvolution. This method of deconvolution produces Delay Time maps, which may be less affected by contrast delay and dispersion than the commonly used time to peak of the residue function maps (Tmax). Processed maps included qualitative parametric maps (including delay time to peak of the residue function (DT), mean transit time (MTT), cerebral blood flow (CBF) and cerebral blood volume (CBV)) and quantitative risk maps based on standard thresholds of CBF < 30 % for core and DT > 3 s for penumbra.

### 2.4 Imaging analysis

All post-processed CTP images from the test and validation cohort were independently reviewed by 6 S neurologists with greater than 5 years of post-Fellowship experience. All clinicians were provided with 3 sets of identical but randomly ordered imaging data (Supplementary Fig. S1). Set one contained non-contrast CT (NCCT) imaging alone. Set two contained NCCT and all qualitative parametric maps including CBV, CBF, MTT and DT. Set three contained NCCT, all qualitative parametric maps and the automated core-penumbra summary map. The neurologists were asked to classify patients in each file as either POCI or non POCI. Imaging data was randomly ordered in each file to prevent any backward comparison of results with previous sets. The neurologists were blinded to the final MRI images and received no clinical information.

### 2.5 Deep learning method

In this study, a Dense Convolutional Network (DenseNet-121) architecture with an Adamax optimizer and Cross Entropy loss function was used. The DenseNet-121 model is part of the MONAI framework (version 1.3.0, python version 3.9). Input images were resized to a 128x128x128 matrix after cropping non-brain data out of the image. The model was trained on the University of Melbourne high performance computing platform using Nvidia A100 GPUs.

To reduce the impact of class imbalance a random selection of patients were excluded from the general cohort (n = 151) to create an analysis cohort with a 1:3 split POCI to Non-POCI split (POCI: 130 to Non-POCI: 390). To bolster the number of non-stroke patients and simulate the real world diagnostic challenge of stroke mimics and transient ischaemic attack; a random sample of non-stroke patients (n = 21) were manually included in the analysis cohort. The analysis cohort was randomly divided into a training (80 %, n = 437), validation (10 %, n = 52) and test (10 %, n = 52) cohort using the train_test_split module using the Python scikit-learn package. Participant allocation was stratified according to lesion size to ensure small lesions were equally represented between groups. DenseNet performance was evaluated on the basis of results from the test cohort.

### 2.6 Statistical analysis

Data processing, analyses and visualisation were performed using Python (version 3.9) using the NumPy, SciPy and Matplotlib packages. Patient characteristics including age, gender, vascular risk factors, baseline NIHSS, treatment and volumetric imaging results were assessed using standard descriptive statistics. Continuous data were assessed for normality using the Shapiro-Wilk test. Non-normally distributed variables were presented as median (interquartile range [IQR]). Univariate relationship of baseline characteristics with POCI cohort versus the general cohort was assessed. Non-normally distributed variables were presented as median (interquartile range [IQR]). The Χ^2^ test was used for categorical variables and the Wilcoxon rank-sum test for non-normally distributed continuous variables. A two tailed P-value of < 0.05 was the cut-off for statistical significance.

Sensitivity, specificity, predictive values, accuracy and the area under the receiver operating characteristic were calculated for clinician assessment of the separate imaging sets (NCCT, NCCT + parametric maps, NCCT + parametric maps + automated core penumbra map) in identifying radiological confirmed POCI on follow up imaging. Optimal clinician classification performance was defined as the combination of imaging which resulted in the highest area under the curve. Good diagnostic accuracy was defined as an AUC > 0.8. Sensitivity and specificity analyses were compared with the Related Samples McNemar Change tests. Agreement between clinicians was evaluated using the Fleiss Kappa statistic. A cut-off of > 0.6 was used to indicate substantial agreement.

### 2.7 Data availabil ity

INSPIRE is a current prospectively recruiting database. Individual patient data from INSPIRE is not publicly available. Individual data can be shared with partners following individual transfer agreements upon request with the corresponding author.

## 3 Results

### 3.1 Patients

**Fig. 1:**
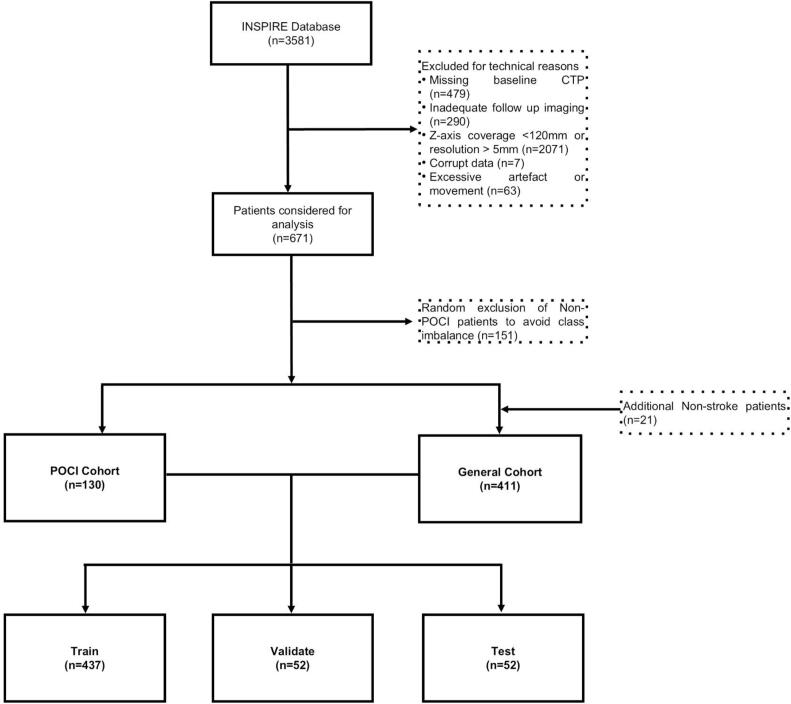
STandards for the reporting of Diagnostic accuracy studies (STARD) flow diagram of patient selection process.

There were 3581 patients from the INSPIRE registry considered for analysis. Five hundred and forty-one patients were included for final analysis. There were 130 patients in the POCI cohort and 411 patients in the general cohort. The general cohort consisted of 28 patients with perforating middle cerebral artery ischaemic stroke, 385 patients with cortical middle cerebral or anterior cerebral artery ischaemic stroke and 36 patients with non-stroke (Bonkhoff et al., 2021). The Exclusions are detailed in the STAndards for the Reporting of Diagnostic accuracy studies (STARD) diagram (Fig. 1).

There were differences in the baseline characteristics between the POCI and general cohorts (Table 1). Compared to the general cohort; POCI patients were more frequently male and less likely to have documented ischaemic heart disease, atrial fibrillation or a 3 month modified Rankin score of 0 to 2 (all p < 0.05). POCI patients had smaller volume lesions on acute (POCI core/penumbra: 1 ml/28 ml, Non-POCI core/penumbra: 7 ml/66 ml, p < 0.05) and follow up imaging (POCI: 4.6 ml, Non-POCI: 11 ml, p < 0.05). There was no significant difference in the acute treatment profile of each cohort.

**Table 1:** Baseline demographic and clinical characteristics of the Posterior circulation infarction and General cohorts.

Characteristics	POCI (n = 130)	General cohort (n = 411)	P-value
Age (median, years)	68 (IQR: 59–76)	71 (IQR: 60–80)	0.05
Female	42 (32.3 %)	188 (45.7 %)	0.01
NIHSS at admission	10 (IQR: 5–18)	11 (IQR: 15–16)	0.64
CTP ischemic core	1 (IQR: 0–3)	7 (IQR: 0–26)	<0.001
CTP penumbra	28 (IQR:3–73.5)	66 (IQR: 7–126)	<0.001
Final infarct volume	4.6 (IQR: 1–22.7)	11 (IQR: 1.5–40.5)	0.02
Treatment None IVT EVT IVT + EVT	36 (28.6 %) 30 (23.8 %) 45 (35.7 %) 15 (11.9 %)	139 (34.1 %) 121 (30.0 %) 99 (24.5 %) 45 (11.1 %)	0.08
Stroke Risk factors Hypertension Hypercholesterolemia Diabetes mellitus Atrial fibrillation Current smoker IHD	78 (65 %) 29 (26.1 %) 32 (27.8 %) 26 (23 %) 24 (21.8 %) 9 (8 %)	229 (61.7 %) 106 (32.4 %) 66 (18.7 %) 130 (36.7 %) 59 (19.5 %) 49 (14.8 %)	0.59 0.26 0.05 0.01 0.71 0.01
3 months mRS			

POCI: Posterior circulation infarction. NIHSS: National Institute of Health Stroke Scale score; CTP: CT Perfusion, IVT: Intravenous thrombolysis; EVT: endovascular thrombectomy; IHD: Ischemic heart disease; mRS; Modified Rankin Score, IQR: Interquartile range.

### 3.2 Clinician classification of posterior circulation infarction

All classification metrics including accuracy, precision, sensitivity, specificity and area under the curve incrementally improved with the inclusion of additional CTP imaging data to NCCT (Table 2). Best mean diagnostic accuracy of all 6 clinicians was seen with a combination of NCCT, parametric maps and the automated core-penumbra map (AUC 0.81). There was variability in individual clinician diagnostic accuracy with an AUC range of 0.73-0.0.86. Details of individual clinician performance are provided in Table 3.

**Table 2:** Clinician performance in classification of Posterior circulation infarction.

Imaging available to clinician	Accuracy	Precision	Sensitivity	Specificity	AUC	Agreement**
NCCT, 95 % CI	0.70 (0.65–0.75)	0.23 (0.09–0.83)	0.09 (0.04–0.14)	0.90 (0.82–0.98)	0.50 (0.43–0.56)	0.17 (0.03–0.30)
NCCT + parametric maps*, 95 % CI	0.85 (0.82–0.88)	0.82 (0.74–0.90)	0.51 (0.43–0.58)	0.96 (0.94–0.98)	0.74 (0.68–0.78)	0.65 (0.47–0.79)
NCCT + parametric maps* + automated core-penumbra map, 95 % CI	0.88 (0.85–0.91)	0.84 (0.72–1.00)	0.65 (0.55–0.74)	0.96 (0.92–1.00)	0.81 (0.74–0.87)	0.73 (0.6–0.82)

*Parametric maps included Cerebral blood flow, Cerebral blood volume, Mean transit time and Delay time. **Agreement as measured by the Fleiss’ Kappa agreement statistic. 95% CI: 95% confidence intervals. NCCT: Non-contrast Computerised tomography of the brain. AUC: Area under the curve.

**Table 3:** Clinician performance based on interpretation of NCCT + parametric maps + automated core-penumbra map.

Clinician	Accuracy	Precision	Sensitivity	Specificity	AUC
1	0.88	1.00	0.54	1.00	0.77
2	0.90	0.90	0.69	0.97	0.83
3	0.90	0.83	0.77	0.95	0.86
4	0.91	1.00	0.65	1.00	0.83
5	0.87	0.75	0.69	0.92	0.81
6	0.82	0.67	0.54	0.91	0.73

AUC: Area under the curve.

**Fig. 2:**
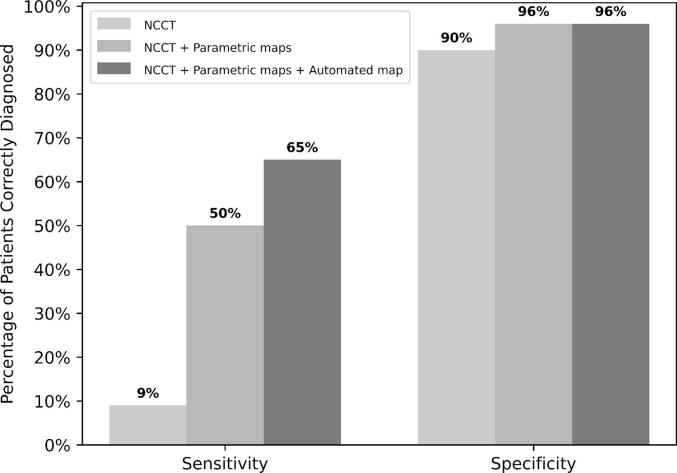
Diagnostic accuracy of Clinicians at classification of Posterior circulation infarction after assessment of Non-contrast CT alone versus Non-contrast CT and Parametric maps versus Non-contrast CT, Parametric maps and Automated core-penumbra map. There is statistically significant improvement in sensitivity with additional CT perfusion data (p < 0.05). There is a statistically significant improvement in specificity with the addition of Parametric maps to NCCT alone. There is no change in specificity with the addition of the Automated core-penumbra map to Non-contrast CT and Parametric maps. NCCT: Non-contrast computerised tomography of the brain. Parametric maps include: Cerebral blood flow, Cerebral blood volume, Mean transit time and Delay time.

There was a statistically significant improvement in sensitivity as more imaging data was added to NCCT with a respective sensitivity of; 0.09 for NCCT alone, 0.51 for NCCT + parametric maps and 0.65 for NCCT + parametric maps + automated core penumbra map (all p < 0.05) (Fig. 2). There was a statistically significant improvement in specificity with the addition of all parametric maps to NCCT (p < 0.05). However, there was no further improvement in specificity when the automated core-penumbra map was added to NCCT and parametric maps.

Clinician agreement incrementally improved with additional imaging data (Table 2). Best clinician agreement was seen with inspection of NCCT + parametric maps + the automated core-penumbra map with a Fleiss-Kappa statistic of 0.73.

Deep learning method for classification of posterior circulation infarction

Eleven models demonstrated good diagnostic accuracy (Table 4). All models demonstrating good diagnostic incorporated at least 2 CT parameters with one of these parameters being either DT or MTT. Results of all analysed DenseNet input combinations are included in Sup. Table S2.

**Table 4:** All data input combinations to the DenseNet model demonstrating good diagnostic accuracy*.

Model Input	Accuracy	Precision	Sensitivity	Specificity	AUC
CBF + DT	0.89	0.82	0.75	0.95	0.85
CBV + DT	0.89	1	0.70	1	0.85
CBV + MTT	0.87	1	0.63	1	0.82
DT + MTT	0.89	1	0.70	1	0.85
**DT + NCCT**	**0.92**	**0.91**	**0.77**	**0.97**	**0.87**
CBF + CBV + DT	0.89	0.82	0.69	0.95	0.82
CBF + DT + MTT	0.89	1	0.70	1	0.85
CBF + DT + NCCT	0.92	1	0.69	1	0.85
CBV + DT + MTT	0.89	1	0.70	1	0.85
CBV + DT + NCCT	0.92	1	0.69	1	0.85
DT + MTT + NCCT	0.90	0.83	0.77	0.95	0.86

*Good diagnostic accuracy defined as an AUC > 0.8. CBF: Cerebral blood flow. CBV: Cerebral blood volume. DT: Delay time. MTT: Mean transit time. Non-contrast Computerised tomography of the brain.

Best model accuracy was seen with the input combination of NCCT and DT maps (AUC 0.87). The sensitivity of this combination was 0.77 which was higher than best mean clinician sensitivity (0.66). Given the spectrum of individual clinician diagnostic accuracy, the outperformance of the model over each clinician varied with an AUC improvement ranging from 0.01 to 0.14. Similarly, the DenseNET improvement in sensitivity ranged from 0 to 0.023 depending on individual clinician result.

**Fig. 3:**
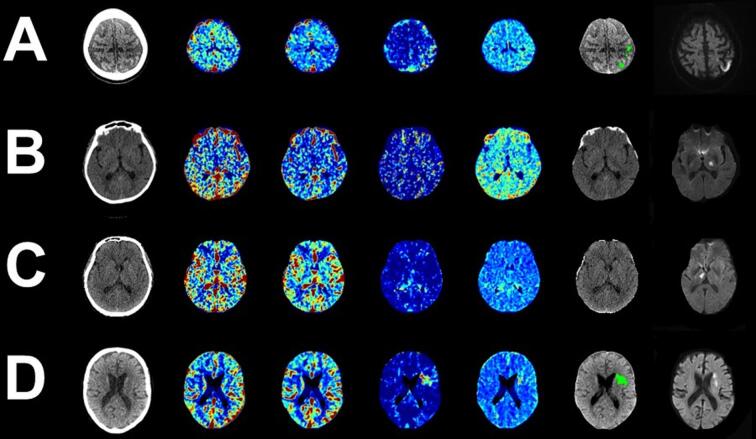
All misclassified patients by the deep learning model in the test cohort. There were three misclassified lesions from the posterior circulation infarction cohort (A-C) and one misclassified lesion from the general cohort (D). Displayed imaging in order of appearance includes: Baseline Non-contrast Computerised tomography, Cerebral blood flow, Cerebral blood volume, Delay time, Mean transit time, Automated core-penumbra map and Follow up Diffusion Weighted Magnetic Resonance imaging.

**Fig. 4:**
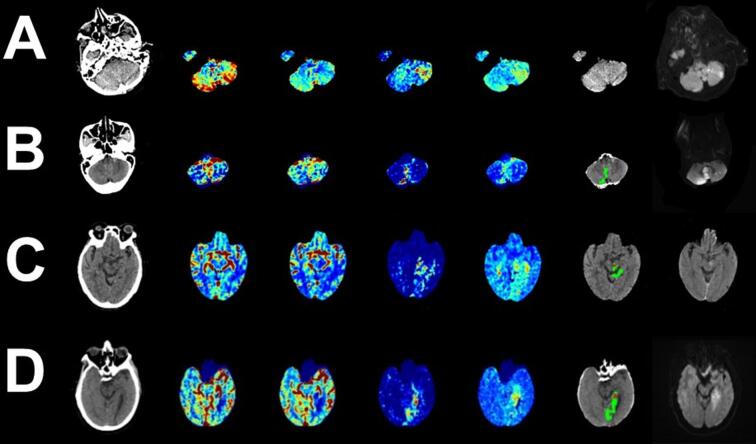
A selection of correctly classified posterior circulation infarction by the deep learning model. Examples include: a left cerebellar stroke not identified by the automated core penumbra map at standard thresholds (A), a bilateral cerebellar stroke (B), a left pontine stroke (C) and a left occipital stroke (D). Displayed imaging in order of appearance includes: Baseline Non-contrast Computerised tomography, Cerebral blood flow, Cerebral blood volume, Delay time, Mean transit time, Automated core-penumbra map and Follow up Diffusion Weighted Magnetic Resonance imaging.

There were 4 misclassified lesions (Sup. Table S3) by the deep learning model. Of the misclassified lesions; there were 3 POCI and 1 non-POCI (Fig. 3). Misclassified lesions were smaller (median volume: 4.8 ml^3^) than correctly classified lesions (median volume: 9.6 ml^3^) and tended to involve subcortical structures such as the thalamus or borderzone territories (Fig. 3). The deep learning model was able to correctly identify a spectrum of POCI, including lesions not detectable at standard automated core-penumbra thresholds (Fig. 4).

## 4 Discussion

Results of this study demonstrate the diagnostic utility of CTP in the classification of acute POCI. Comprehensive assessment of NCCT, parametric maps and the automated core-penumbra map resulted in best diagnostic accuracy and best agreement amongst clinicians. Both expert assessment and an automated deep learning approach demonstrated good diagnostic accuracy. The DenseNET model had better diagnostic accuracy and improved sensitivity compared to best mean clinician result using multimodal CT imaging alone. This degree of superiority varied according to the individual clinician.

While major guidelines recommend multimodal imaging with NCCT and CTA in all stroke patients, CTP is often considered optional (Powers et al., 2018). MRI is considered the gold standard for diagnosis of stroke, in particular POCI (Muir et al., 2006). Its utility in acute stroke is hampered by limited 24/7 availability and inability to be performed in patients with metal foreign bodies such as pacemakers. CTP does not face these challenges. Diagnostic accuracy of MRI is limited in acute POCI with up to 31 % of patients having a false negative result on diffusion weighted MRI within 24 h of symptoms (Oppenheim et al., 2000).

Two prevailing criticisms of CTP have been the lack of data demonstrating additional diagnostic benefit and limited utility in POCI. This study aimed to address these concerns. There are few studies of CTP in POCI (Edwards et al., 2021). Current thresholds used by commercial packages for delineation of core and penumbra have been derived from studies of anterior circulation stroke which are suboptimal in POCI (Edwards et al., 2023). CTP has demonstrated clear clinical utility in POCI particularly with expert inspection of parametric maps (Ostman et al., 2020). Osteman et al reported an 40 % improvement in sensitivity with comprehensive expert review of NCCT, parametric maps and automated core penumbra maps compared to NCCT alone. This study supports these results and confirms the diagnostic utility of comprehensive imaging assessment of CTP maps for POCI classification. Even at standard thresholds, there was a 56 % increase in sensitivity and improvement in AUC from 0.50 to 0.81 with inspection of all CT imaging compared to NCCT alone. Specificity remained static or improved with additional maps; thereby implying there was no increase in false positive rate despite improved detection of POCI. These findings have important clinical implications particularly for centres in which CTP is not standard of care. The results of this study suggest routine inspection of mCT including CTP parametric and automated core-penumbra map enhance detection of POCI with the potential to improve access to acute reperfusion therapies and stroke care.

A major challenge in the rapid and accurate diagnosis of POCI is access to specialist services and inter-rater agreement. Untreated POCI has high rates of mortality and morbidity, which may be ameliorated with early diagnosis and reperfusion therapies (Machner et al., 2021). Up to 20–35 % of patients presenting to emergency services with POCI symptoms receive incorrect diagnoses (Machner et al., 2021, Nham et al., 2022). Studies have shown access to specialist neurology (Borgwardt and (Neurologe) DS, 2014) and imaging services (Barber et al., 2000, Wang, 2010) improves diagnostic accuracy. Many settings have limited access to this finite expertise (Edwards et al., 2020). Computer aided diagnostic methods present an attractive method to overcoming these skills shortages. In this study, both comprehensive assessment of CTP imaging and a deep learning approach was superior to assessment of NCCT alone. The deep learning approach was superior to the aggregate clinician result. Most of the diagnostic improvement was through enhanced detection of POCI represented by a 12 % improvement in sensitivity. At an individual expert level, this improvement was less pronounced and varied from 0-23 %. Previous studies have shown similar variability in assessment of acute stroke imaging amongst expert readers (Barber et al., 2000). This variability is even more pronounced with less experienced clinicians(Meyer et al., 2009). Hybrid computer aided clinician assessment of stroke imaging has been shown to standardise stroke detection(Li et al., 2020). Comprehensive review of CTP imaging improved agreement amongst clinician assessment. Adding the DenseNet classification tool to expert clinician assessment may help to further reduce the variability in diagnostic accuracy and sensitivity seen in this study. The improvement is likely to be even more pronounced in resource limited settings.

There are several limitations to the present study. The model was trained, validated and tested using a single (albeit multi-centre and multi-national) dataset. Dividing the single dataset resulted in a restricted test cohort on which the model was evaluated. Robust subgroup analysis was limited given the small number of misclassified patients. Future studies should validate the model in a separate independent prospective cohort of real-world patients to determine its true clinical applicability. This study used one commercial package for processing of CTP maps. Given the considerable variability in CTP maps produced by software vendors (Bivard et al., 2013), this may impact the performance of the model and limit its applicability to maps produced by other software packages, which may be less accurate. This study included only scans with a z-axis range of at least 120 mm. This is less than the range of newer 320 detector scanners capable of brain acquisition. This limited scan range may reduce the performance of both clinicians and the model. CT angiography data were not included in this study. This is likely to reduce the diagnostic accuracy of both clinicians and the DenseNET model. Posterior circulation subregions were not specifically analysed. Studies have shown that accuracy of CTP varies by anatomical subregion[26] with lower diagnostic certainty outside of the cerebral cortex or cerebellum. Analysis of these regions should be the direction of future studies.

## 5 Conclusion

Comprehensive inspection of non-contrast CT, parametric maps and the automated core-penumbra map improves diagnostic accuracy and agreement amongst expert readers. At an aggregate level, a DenseNet approach to POCI classification has superior performance to experts, however this varies at an individual clinician level. The majority of improvement is represented by increased sensitivity in the detection of true strokes. Future studies focusing on computer aided assessment of POCI may help to reduce clinician variability, improve diagnosis and broaden access to acute stroke therapies, particularly in resource limited settings.

Funding.

This research is supported by an Australian Government Research Training Program (RTP) Scholarship.

## CRediT authorship contribution statement

**Leon S. Edwards:** . **Milanka Visser:** Writing – review & editing, Writing – original draft, Methodology, Investigation, Formal analysis, Data curation, Conceptualization. **Cecilia Cappelen-Smith:** Writing – review & editing, Writing – original draft, Supervision, Formal analysis. **Dennis Cordato:** Writing – review & editing, Writing – original draft, Supervision, Formal analysis, Conceptualization. **Andrew Bivard:** Writing – review & editing, Writing – original draft, Supervision, Methodology, Investigation, Formal analysis, Data curation, Conceptualization. **Leonid Churilov:** Writing – review & editing, Writing – original draft. **Christopher Blair:** Writing – review & editing, Writing – original draft, Formal analysis. **James Thomas:** Writing – review & editing, Writing – original draft, Formal analysis. **Angela Dos Santos:** Writing – review & editing, Writing – original draft, Formal analysis. **Longting Lin:** Writing – review & editing, Writing – original draft, Data curation, Conceptualization. **Chushuang Chen:** Writing – review & editing, Writing – original draft, Methodology, Investigation. **Carlos Garcia-Esperon:** Writing – review & editing, Writing – original draft. **Kenneth Butcher:** Writing – review & editing, Writing – original draft. **Tim Kleinig:** Writing – review & editing. **Phillip MC Choi:** Writing – review & editing. **Xin Cheng:** Writing – review & editing. **Qiang Dong:** Writing – review & editing. **Richard I. Aviv:** Writing – review & editing, Conceptualization. **Mark W. Parsons:** Writing – review & editing, Writing – original draft, Supervision, Resources, Formal analysis, Data curation, Conceptualization.

## Declaration of competing interest

The authors declare that they have no known competing financial interests or personal relationships that could have appeared to influence the work reported in this paper.

## Data Availability

Data will be made available on request.

## References

[b0005] Albers G.W., Marks M.P., Kemp S., Christensen S., Tsai J.P., Ortega-Gutierrez S. (2018). Thrombectomy for Stroke at 6 to 16 Hours with Selection by Perfusion Imaging. N Engl J Med..

[b0010] Arch A.E., Weisman D.C., Coca S., Nystrom K.V., Wira C.R., Schindler J.L. (2016). Missed Ischemic Stroke Diagnosis in the Emergency Department by Emergency Medicine and Neurology Services. Stroke..

[b0015] Bamford J., Sandercock P., Dennis M., Burn J., Warlow C. (1991). Classification and natural history of clinically identifiable subtypes of cerebral infarction. Lancet..

[b0020] Barber P.A., Demchuk A.M., Zhang J., Buchan A.M. (2000). Validity and reliability of a quantitative computed tomography score in predicting outcome of hyperacute stroke before thrombolytic therapy. ASPECTS Study Group. Alberta Stroke Programme Early CT Score. Lancet..

[b0025] Bivard A., Levi C., Spratt N., Parsons M. (2013). Perfusion CT in acute stroke: a comprehensive analysis of infarct and penumbra. Radiology..

[b0030] Bonkhoff A.K., Xu T., Nelson A., Gray R., Jha A., Cardoso J. (2021). Reclassifying stroke lesion anatomy. Cortex..

[b0035] Borgwardt RS, (Neurologe) DS. Referral and Final Diagnoses of Patients Assessed in an Academic Vertigo Center. 2014. 10.3389/fneur.2012.00169 PMC3508265 23226141

[b0040] Edwards L.S., Blair C., Cordato D., McDougall A., Manning N., Cheung A. (2020). Impact of interhospital transfer on patients undergoing endovascular thrombectomy for acute ischaemic stroke in an Australian setting. BMJ Neurology Open..

[b0045] Edwards L.S., Cappelen-Smith C., Cordato D., Bivard A., Churilov L., Parsons M.W. (2021). Review of CT perfusion and current applications in posterior circulation stroke. Vessel plus..

[b0050] Edwards L.S., Cappelen-Smith C., Cordato D., Bivard A., Churilov L., Lin L. (2023). Optimal CT perfusion thresholds for core and penumbra in acute posterior circulation infarction. Front Neurol..

[b0055] Heo J, Yoon JG, Park H, Kim YD, Nam HS, Heo JH. Machine Learning–Based Model for Prediction of Outcomes in Acute Stroke. Stroke. 2019 [cited 19 Jul 2023]. doi:10.1161/STROKEAHA.118.024293. 10.1161/STROKEAHA.118.024293 30890116

[b0060] Huang G., Liu Z., Van Der Maaten L., Weinberger K.Q. (2017). 2017 IEEE Conference on Computer Vision and Pattern Recognition (CVPR). IEEE.

[b0065] Kim J.-T., Park M.-S., Choi K.-H., Kim B.J., Han M.-K., Park T.H. (2017). Clinical Outcomes of Posterior Versus Anterior Circulation Infarction With Low National Institutes of Health Stroke Scale Scores. Stroke..

[b0070] Lakshmi K.S., Sargunam B. (2024). Exploration of AI-powered DenseNet121 for effective diabetic retinopathy detection. Int Ophthalmol..

[b0075] Lee H., Lee E.-J., Ham S., Lee H.-B., Lee J.S., Kwon S.U. (2020). Machine Learning Approach to Identify Stroke Within 4.5 Hours. Stroke..

[b0080] Li L., Chen Y., Bao Y., Jia X., Wang Y., Zuo T. (2020). Comparison of the performance between Frontier ASPECTS software and different levels of radiologists on assessing CT examinations of acute ischaemic stroke patients. Clin Radiol..

[b0085] Ma H., Campbell B.C.V., Parsons M.W., Churilov L., Levi C.R., Hsu C. (2019). Thrombolysis Guided by Perfusion Imaging up to 9 Hours after Onset of Stroke. N Engl J Med..

[b0090] Machner B., Choi J.H., Neumann A., Trillenberg P., Helmchen C. (2021). What guides decision-making on intravenous thrombolysis in acute vestibular syndrome and suspected ischemic stroke in the posterior circulation?. J Neurol..

[b0095] Meyer R.E., Nickerson J.P., Burbank H.N., Alsofrom G.F., Linnell G.J., Filippi C.G. (2009). Discrepancy rates of on-call radiology residents’ interpretations of CT angiography studies of the neck and circle of Willis. AJR Am J Roentgenol..

[b0100] Muir K.W., Buchan A., von Kummer R., Rother J., Baron J.-C. (2006). Imaging of acute stroke. Lancet Neurol..

[b0105] Nham B., Reid N., Bein K., Bradshaw A.P., McGarvie L.A., Argaet E.C. (2022). Capturing vertigo in the emergency room: three tools to double the rate of diagnosis. J Neurol..

[b0110] Nogueira R.G., Jadhav A.P., Haussen D.C., Bonafe A., Budzik R.F., Bhuva P. (2018). Thrombectomy 6 to 24 Hours after Stroke with a Mismatch between Deficit and Infarct. N Engl J Med..

[b0115] Oppenheim C., Stanescu R., Dormont D., Crozier S., Marro B., Samson Y. (2000). False-negative diffusion-weighted MR findings in acute ischemic stroke. AJNR Am J Neuroradiol..

[b0120] Ostman C., Garcia-Esperon C., Lillicrap T., Tomari S., Holliday E., Levi C. (2020). Multimodal Computed Tomography Increases the Detection of Posterior Fossa Strokes Compared to Brain Non-contrast Computed Tomography. Front Neurol..

[b0125] Pagola J., Ribo M., Alvarez-Sabin J., Rubiera M., Santamarina E., Maisterra O. (2011). Thrombolysis in anterior versus posterior circulation strokes: timing of recanalization, ischemic tolerance, and other differences. J Neuroimaging..

[b0130] Paul N.L.M., Simoni M., Rothwell P.M. (2013). Oxford Vascular Study. Transient isolated brainstem symptoms preceding posterior circulation stroke: a population-based study. Lancet Neurol..

[b0135] Powers W.J., Rabinstein A.A., Ackerson T., Adeoye O.M., Bambakidis N.C., Becker K. (2018). 2018 Guidelines for the Early Management of Patients With Acute Ischemic Stroke: A Guideline for Healthcare Professionals From the American Heart Association/American Stroke Association. Stroke..

[b0140] Sarraj A., Medrek S., Albright K., Martin-Schild S., Bibars W., Vahidy F. (2015). Posterior circulation stroke is associated with prolonged door-to-needle time. Int J Stroke..

[b0145] Schulz U.G., Fischer U. (2017). Posterior circulation cerebrovascular syndromes: diagnosis and management. J Neurol Neurosurg Psychiatry..

[b0150] Sharon M., Boyle K., Yeung R., Zhang L., Symons S.P., Boulos M.I. (2016). The predictive value of a targeted posterior fossa multimodal stroke protocol for the diagnosis of acute posterior ischemic stroke. Neurovascular Imaging..

[b0155] Tarnutzer A.A., Lee S.-H., Robinson K.A., Wang Z., Edlow J.A., Newman-Toker D.E. (2017). ED misdiagnosis of cerebrovascular events in the era of modern neuroimaging: A meta-analysis. Neurology..

[b0160] Thomalla G., Simonsen C.Z., Boutitie F., Andersen G., Berthezene Y., Cheng B. (2018). MRI-Guided Thrombolysis for Stroke with Unknown Time of Onset. N Engl J Med..

[b0165] Urinbayev K., Orazbek Y., Nurambek Y., Mirzakhmetov A., Varol H.A. (2020). End-to-End Deep Diagnosis of X-ray Images. Conf Proc IEEE Eng Med Biol Soc..

[b0170] Wang M.L. (2010). Discrepancy rates of on-call radiology residents’ interpretations of CT angiography studies of the neck and circle of Willis. AJR Am J Roentgenol..

