# Supplementary material for: A deep learning approach versus expert clinician panel in the classification of posterior circulation infarction
Source: Neuroimage Clin. 2025 Jan 12;45:103732. doi: 10.1016/j.nicl.2025.103732 (PMC11786091; doi:10.1016/j.nicl.2025.103732)
Supplement: Supplementary Data 1 [file mmc1.docx]

**Supplement**

**Supplementary table 1 - Acute CT perfusion acquisition protocols for the nine INSPIRE sites included in this study**

| **Site** | **CT Scanner** | **Acquisitions** | **Coverage** | **Contrast** |
| --- | --- | --- | --- | --- |
| Gosford hospital, Australia | GE lightspeed VCT (64 detectors) | 2 slabs; 19 acquisitions in 54 seconds for each slab | 80 mm | 45 mL of contrast (Ultravist 370) injected at 6 mL/s for each aquisition |
| Royal Adelaide Hospital, Australia | SIEMENS SOMATOM Definition AS+ (128 detectors | 29 acquisitions in 58 seconds | 96 mm | 40 mL of contrast (Ultravist 370) injected at 6 mL/s |
| The Queen Elizabeth Hospital, Australia | Toshiba Aquilion one (320 detectors) | 19 acquisitions in 60 seconds | 160 mm | 40 mL of contrast (Ultravist 370) injected at 6 mL/s |
| Box Hill Hospital, Australia | Toshiba Aquilion one (320 detectors) | 19 acquisitions in 60 seconds | 160 mm | 40 mL of contrast (Ultravist 370) injected at 6 mL/s |
| John Hunter Hospital, Australia | Toshiba Aquilion one (320 detectors) | 19 acquisitions in 60 seconds | 160 mm | 40 mL of contrast (Ultravist 370) injected at 6 mL/s |
| The Second Affiliated Hospital of Zhejiang University, China | SIEMENS SOMATOM Definition Flash (256 detectors) | 25 acquisitions in 62 second | 100 mm | 15 mL of contrast agent (Ultravist 370) injected at 4mL/s |
| Huashan Hospital, China | Philips Brilliance iCT (256 detectors) | 12 acquisitions in 54 seconds; shuttle mode | 125 mm | 40 mL of contrast agent (Ultravist 370) injected at 5 mL/s |
| Baotou Central Hospital, China | SIEMENS SOMATOM Definition Flash (256 detectors) | 17 acquisitions in 72 second | 150 mm | 40 mL of contrast agent (Ultravist 350) injected at 5mL/s |
| Sunnybrook Medical Centre, Canada | GE lightspeed VCT (64 detectors) | 51 acquisitions in 135 seconds | 40 mm | 0.7 mL/kg (maximum to 90 mL) of iodinated contrast agent (Omnipaque, 300 mg iodine/mL) injected at 2-4 mL/seconds |

INSPIRE: International stroke perfusion imaging registry.

**Supplementary table 2 - Results of all data input combinations for the DenseNet model**

| **Model Input** | **Accuracy** | **Precision** | **Sensitivity** | **Specificity** | **AUC** |
| --- | --- | --- | --- | --- | --- |
| CBF | 0.85 | 0.78 | 0.54 | 0.95 | 0.75 |
| CBV | 0.79 | 1 | 0.15 | 1 | 0.58 |
| DT | 0.65 | 0.22 | 0.15 | 0.82 | 0.49 |
| MTT | 0.56 | 0.25 | 0.39 | 0.62 | 0.51 |
| NCCT | 0.75 | - | 0 | 1 |  |
| CBF + CBV | 0.89 | 0.89 | 0.62 | 0.97 | 0.8 |
| CBF + DT | 0.89 | 0.82 | 0.75 | 0.95 | 0.85 |
| CBF + MTT | 0.79 | 0.58 | 0.56 | 0.87 | 0.72 |
| CBF + NCCT | 0.79 | 0.67 | 0.42 | 0.95 | 0.69 |
| CBV + DT | 0.89 | 1 | 0.70 | 1 | 0.85 |
| CBV + MTT | 0.87 | 1 | 0.63 | 1 | 0.82 |
| CBV + NCCT | 0.79 | 0.60 | 0.52 | 0.90 | 0.71 |
| DT + MTT | 0.89 | 1 | 0.70 | 1 | 0.85 |
| DT + NCCT | 0.92 | 0.91 | 0.77 | 0.97 | 0.87 |
| CBF + CBV + DT | 0.89 | 0.82 | 0.69 | 0.95 | 0.82 |
| CBF + CBV + MTT | 0.81 | 0.90 | 0.58 | 0.90 | 0.74 |
| CBF + DT + MTT | 0.89 | 1 | 0.70 | 1 | 0.85 |
| CBF + DT + NCCT | 0.92 | 1 | 0.69 | 1 | 0.85 |
| CBV + DT + MTT | 0.89 | 1 | 0.70 | 1 | 0.85 |
| CBV + DT + NCCT | 0.92 | 1 | 0.69 | 1 | 0.85 |
| DT + MTT + NCCT | 0.90 | 0.83 | 0.77 | 0.95 | 0.86 |

CBF: Cerebral blood flow. CBV: Cerebral blood volume. DT: Delay time. MTT: Mean transit time. Non-contrast Computerised tomography of the brain.

**Supplementary table 3 - Characteristics of incorrectly classified patients by the Deep learning model in the test cohort**

| **Patient** | **Group** | **DWI lesion volume, ml^3^** | **CBF < 30%, ml^3^** | **DT > 3 secs, ml^3^** | **Occlusion location** |
| --- | --- | --- | --- | --- | --- |
| 1 | POCI | 10.0 | 0.1 | 12.0 | Left PCA |
| 2 | POCI | 3.6 | 2.0 | 4.0 | None |
| 3 | POCI | 2.0 | 0.0 | 0.0 | None |
| 4 | General | 5.9 | 0.1 | 22.2 | Left MCA |

DWI: Diffusion weighted imaging, DT: Delay time, CBF: Relative cerebral blood flow, Secs: seconds, ml^3^: cubic millimetres, PCA: posterior cerebral artery, MCA: middle cerebral artery.

**Supplementary Figure 1 - A case example of a Posterior circulation infarction and the data contained within the three imaging sets provided to the expert readers. Set 1 contained non-contrast CT data (A). Set 2 contained non-contrast CT and parametric data including cerebral blood volume, cerebral blood flow, delay time and mean transit time data (B). Set 3 contained non-contrast CT, parametric data and the automated core-penumbra map (C).**

**
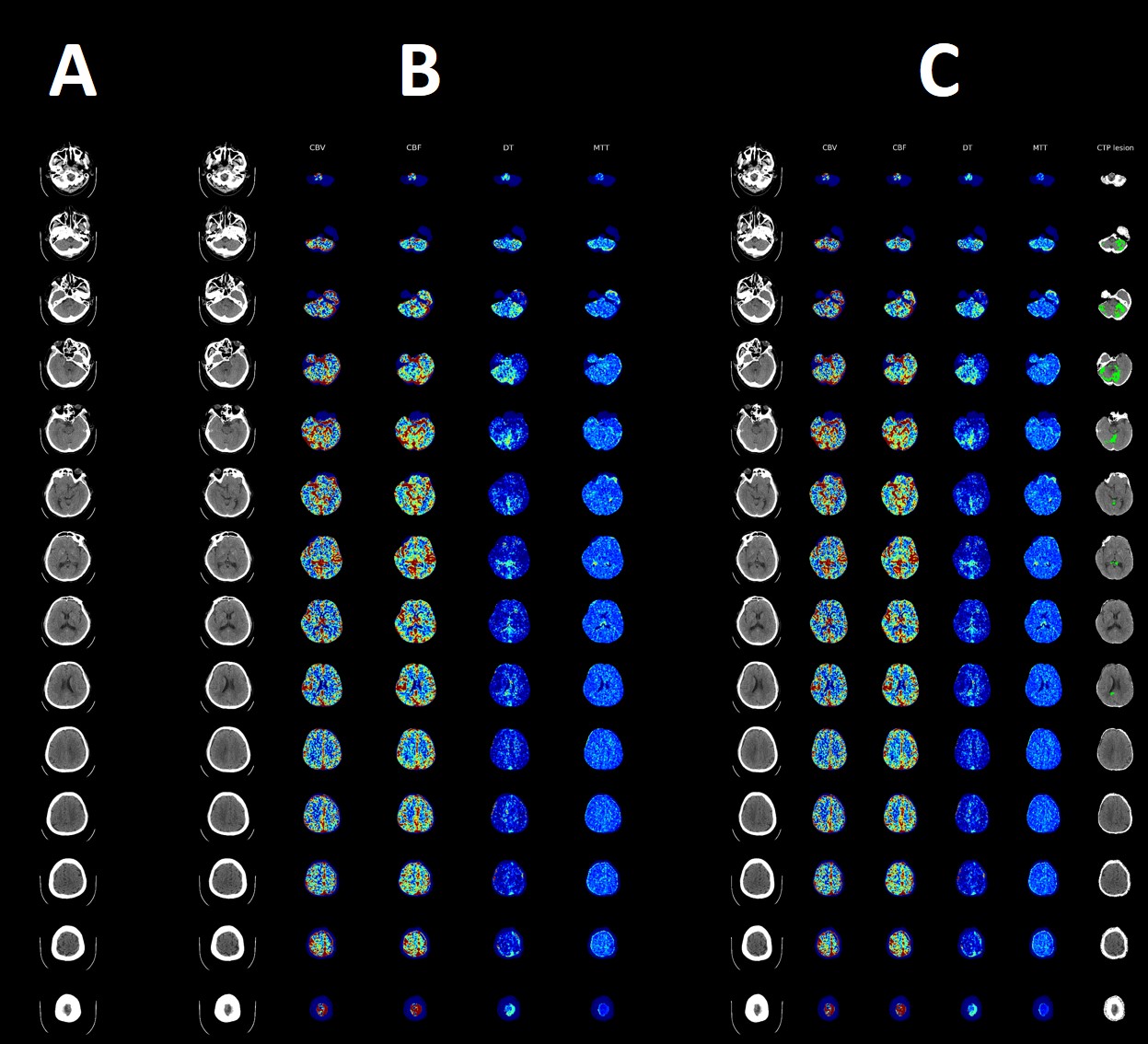
**

**Appendix**

**Appendix: INSPIRE Study Group Co-Investigators**

| **Name** | **Location** | **Role** | **Contribution** |
| --- | --- | --- | --- |
| Ferdinand Miteff, MD | John Hunter Hospital, Newcastle, Australia | Site Investigator | Data collection |
| Congguo Yin, MD | Hangzhou First Hospital, Zhejiang University School of Medicine, Hangzhou, China | Site Investigator | Data collection |
| Peng Wang, MD | Zhejiang Provincial People’s Hospital, Hangzhou, China | Site Investigator | Data collection |
| Yu Geng, MD | Zhejiang Provincial People’s Hospital, Hangzhou, China | Site Investigator | Data collection |
| Xu Zhang, MD | The First Affiliated Hospital of Wenzhou Medical University, Wenzhou, | Site Investigator | Data collection |
| Xuezhi Yang, MD | The First Affiliated Hospital of Wenzhou Medical University, Wenzhou, China | Site Investigator | Data collection |
| Weiwen Qiu, MD | Lishui People’s Hospital, Lishui, China | Site Investigator | Data collection |
| Qi Fang, MD | The First Affiliated Hospital of Soochow University, Soochow, China | Site Investigator | Data collection |
| Yi Sui, PhD, MD | The First People’s Hospital of Shenyang, Shenyang, China | Site Investigator | Data collection |
| Wenhuo Chen, MD | Zhangzhou Municipal Hospital, Zhangzhou, China | Site Investigator | Data collection |
| Gang Li, PhD, MD | Shanghai East Hospital, Tongji University School of Medicine, Shanghai, China | Site Investigator | Data collection |
